# Voluntary contractions underestimate peak muscle activity in drop jumps

**DOI:** 10.1080/23335432.2025.2518343

**Published:** 2025-06-09

**Authors:** David Alan Phillips, Skylar Paletta, Michael Perlet

**Affiliations:** a College of Health, Program in Kinesiology, Oregon State University-Cascades, Bend, Oregon; b Department of Kinesiology, Montclair state University, Montclair, New Jersey, USA

**Keywords:** Electromyography, drop jump, normalization lower extremity

## Abstract

Maximal voluntary isometric contractions (MVIC) are a common method to normalize electromyographic amplitude into standardized units of %MVIC. However, in 60% of drop jump research using an MVIC in 2018–2023, supramaximal activation or activation greater than 100% MVIC occurred. Therefore, MVICs may not be representative of peak muscle activation, leading to erroneous interpretation of muscle activation. The purpose of this study is to quantify EMG normalization difference in drop jump landings. Sixteen (10 M, 6F) participants were recruited for the study. MVICs were recorded from nine lower extremity muscles and this activation compared to the maximal activation recorded from 10 drop jump trials. The MVIC significantly underestimated maximum activation by 71%–140% in one-sample *t*-tests, for the rectus femoris (*p* = 0.002), vastus medialis (*p* < 0.001), medial gastrocnemius (*p* = 0.002), lateral gastrocnemius (*p* = 0.002), tibialis anterior (*p* = 0.02), and gluteus maximus (*p* = 0.03). The one-sample t-tests were not statistically significant for the remaining muscles with the data containing significant variability. Our data quantifies EMG normalization underestimate and supports the status in the literature where normalization with MVICs will underestimate maximal muscle activation in drop jump movements.

## Introduction

Electromyography (EMG) is a tool used to measure muscle activity in athletic lower extremity movements. This is often done in combination with kinematic and kinetic measures to evaluate the mechanics of the motion. Researchers study these motions to improve performance, reduce injury rates, and develop intervention strategies. Electromyography, unlike kinematic and kinetic measures, presents its units on an arbitrary scale measured in voltage and is influenced by a range of electrophysiological variables (Konrad 2005).

Therefore, to interpret EMG amplitude and compare between individuals or muscles, researchers must normalize the EMG signal to a reference value from the same muscle. The reference value must be obtained from each individual’s muscle using the same procedure. For instance, one method to measure the maximum voluntary isometric contraction (MVIC) muscle activity from the biceps femoris, is the participant lies prone, with the knee flexed to 90°. The researcher or external device then applies a force at the ankle to resist knee flexion while the participant forcefully flexes the knee joint (Hsu et al. 2006). The EMG amplitude is then expressed as a percentage of this reference value. Generally, the reference value is obtained through a maximal voluntary isometric contraction (MVIC), a maximum value measured during a dynamic activity, comparison to a set submaximal load or activity, or with M-wave max amplitude (among others) (Halaki and Gi 2012; Besomi et al. 2020). Normalization to the MVIC and maximum activity value are most common in drop jump research as they are considered valid for between participant and between muscle comparisons (Besomi et al. 2020).

In lower extremity jump research, there is no standardized approach to EMG normalization. However, when using MVIC to normalize EMG amplitude, the maximum activity observed during the activity may be greater than measured during the MVIC resulting in values greater than 100% MVIC or a supramaximal activation. These supramaximal EMG amplitude recordings may arise due to the position the MVIC is recorded in (Contreras et al. 2015; Schwartz et al. 2020), contraction type (Enoka 1996; Grabiner and Owings 2002), participant motivation/encouragement (Mcnair et al. 1996), movement velocity (Ball and Scurr 2010), stretch shortening cycle augmentation (Trimble et al. 2000), and/or muscle crosstalk (Chapman et al. 2010). In a comparison of normalization methods in sprinting and jumping, MVIC generally underestimated maximal activation across all muscles in the lower extremity (Suydam et al. 2017) but this study kept the measures in raw millivolts so the difference between methods is not represented in interpretable units. In our own literature review of the past 5 years of literature (January 2018–January 2023, see supplemental material), we identified 139 studies using EMG amplitude in jumping, cutting, and landing research. Of these, 48 (34.5%) used MVIC, 49 (35%) used an activity max, and 42 (30%) used alternative approaches (or none) to normalize EMG amplitude. We noted greater than 100% activation in 29 (60.4%) of the studies that used MVIC to normalize the EMG data. Seventeen of the studies (35.4%) data reporting data as %MVIC did not allow for sufficient interpretation to determine if supramaximal activation had occurred. Only two studies (4.1%) were determined not to have supramaximal activation within the reported data. Supramaximal activation when using MVIC to normalize EMG amplitude is therefore prevalent in current literature even with existing contraindicating recommendations for high velocity movements (Ball and Scurr 2013). This presents difficulties in using MVIC as a comparison metric and a potential threat to EMG data validity. This may be particularly problematic when research seek to investigate activation ratios between muscles where the magnitude of one muscle may exceed 100%, while the other may not.

The purpose of the study was to quantify EMG normalization difference with an MVIC during a drop jump movement. We hypothesize that an MVIC will underestimate maximum muscle activity across all muscles in a drop jump landing.

## Materials and methods

### Participants

Sixteen participants (10 males and 6 females, age = 22.8 ± 1.4 years, mass = 74 ± 14 kg, height = 173 ± 9.7 cm) completed the single group measure study in a controlled laboratory setting. Participants were excluded if they had a history of a severe traumatic injury of the lower extremity – defined by any pain, or any diagnosed neurological condition affecting motor performance. Before their participation, each participant read and signed a written informed consent form, in accordance with Montclair State University’s Institutional Review Board.

We performed an *a priori* power analysis using a large effect size = 1.0, alpha = 0.05, and power = 0.8 in a one-sample *t*-test to determine the sample size (G*Power, 3.1.9.4) based on pilot testing within the research team. The *a priori* power analysis indicated that a sample of 10 participants would be sufficient to find differences in the dependent variable, max %MVC measured during the drop jump compared to 100% activity maximum for each muscle. The authors opted to recruit *n =* 12 in case of greater than anticipated variability. This sample size is consistent with previous research (Trimble et al. 2000). The independent variable was the normalization method, MVIC, or activity max set at 100%.

### Study design and procedures

After the informed consent process, the sites of all electrode placements were shaved (if applicable) and cleaned using alcohol. The electrode placement sites were gastrocnemius medial and lateral heads, tibialis anterior, medial and lateral hamstring, rectus femoris, vastus medialis oblique, gluteus medius, and gluteus maximus muscles (Hermens et al. 1997; Cram et al. 1998). The electrodes were attached with double-sided tape and wrapped with self-adhesive bandages to maintain contact during the dynamic jumping movements.

The participants were instructed on the general MVIC procedure. In each MVIC, the participant was verbally encouraged to rapidly apply maximum force against an external resistance and maintain the force for 5 s, as per a timer, before relaxing via a cue from the researcher. The participant was allowed submaximal attempts for each muscle group before three maximal trials were collected with a two-minute rest period between each attempt. For each participant, the order of the muscles tested was randomized using the randomize function in Microsoft Excel. The specific positioning for each muscle’s MVIC was according to previous literature and summarized in Table 1. Electromyographic sensors were verified to be receiving a signal, with the participant performing motions targeting the muscles during and immediately after each MVIC recording.

**Table 1.** List of muscles and testing position for the maximal voluntary isometric contraction.

Muscle	MVIC Test Position
Gluteus Maximus	Lying prone, knee flexed 90 degrees (Contreras et al. 2015)
Gluteus Medius	Side lying contralateral from tested side (Bernard et al. 2017)
Rectus Femoris	Seated, hip & knee flexed to 90 degrees (Purkayastha et al. 2006)
Vastus Medialis	Seated, hip & knee flexed to 90 degrees (Purkayastha et al. 2006)
Semitendinosus	Prone, knee flexed to 60 degrees (Hsu et al. 2006)
Biceps Femoris	Prone, knee flexed to 60 degrees (Hsu et al. 2006)
Tibialis Anterior	Seated, hip & knee flexed to 90 degrees, ankle neutral (de Oliveira Sousa et al. 2007)
Medial & Lateral Gastrocnemius	Seated knee flexed to 30 degrees, dorsiflexed 15 degrees (Albertus-Kajee et al. 2011)

Following all MVICs, the participants were instructed on the drop jump procedure and observed the movement demonstration by a researcher. A 30-cm-high box for all participants was placed at a distance of 50% of the participant’s height from the back edge of a force plate. The participant was instructed to jump from the box with both feet simultaneously so that each foot would land on an individual force plate and then jump as high and explosively into the air with both feet landing back on the individual force plates and to stick the landing. The participant was given as many practice attempts as needed to perform the movement correctly. After the practice attempts, 10 successful trials were recorded with a minimum of 30 s rest between each trial.

A wireless surface electromyography (EMG) system (Trigno Sensor System, Delsys Inc., Natick, MA, USA: interelectrode distance = 10 mm, 80 dB common mode rejection rate) was used to record and process muscle activity synchronously with the two force plates (Bertec Corporation, Columbus, OH) at 2000 hz using Motion Monitor. The EMG data were Fourier band pass filtered 20–500 hz (zero lag) (De Luca et al. 2010) and smoothed using a 50 ms root mean square sliding window. Data from the sensors placed on the right side was used for all analyses. The EMG data from the drop jumps are normalized using the highest value recorded from the three MVIC trials to represent the EMG data recorded during the drop jump as a %MVIC. The drop jump that elicited the highest activation was used in the analysis. All trials were screened for artifacts with channels containing large magnitude, single spikes removed from the analysis (the final number of participants (*n*) included in the analysis for each muscle is reported in results after the *p* value.).

### Statistical analysis

A one-sample *t*-test was used to compare maximum values recorded during the drop jump normalized to %MVIC to a set value of 100% for each muscle. If using the maximum activity value to normalize the EMG data, the highest recorded value will be 100% in all cases since it was recorded during the activity. Normal distribution of the data was assessed with a Shapiro–Wilk test. If the data were not normally distributed, the comparisons were also run with a one-sample Wilcoxon Ranked Sign test to confirm any significant observations.

## Results

**Figure 1.**
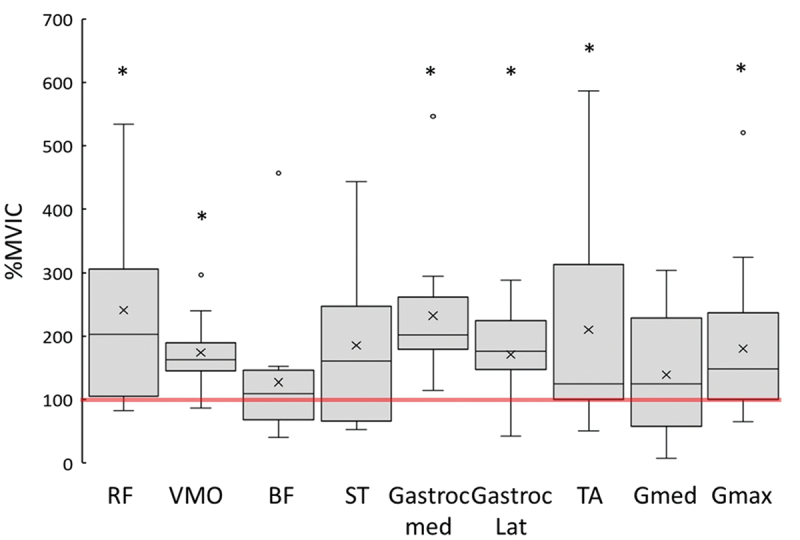
Spread of maximum EMG amplitude measured during the drop jump and normalized to %MVIC. *indicates significantly different from 100% normalized activity max, circles are outliers > 1.5× the interquartile range, x indicates mean, and center line is the median. RF, rectus femoris, VMO, vastus medialis oblique, BF, biceps femoris, ST, semitendinosus, Gastroc med, medial gastrocnemius, Gastroc lat, lateral gastrocnemius, Gmed, gluteus medius, Gmax, gluteus maximus.

Only the rectus femoris and vastus medialis muscle followed a normal distribution (*p* > .05). A significant difference from 100% MVIC was observed for the rectus femoris (*Mean (M)* = 241% ± 140, *p* = .002, *n* = 15), the vastus medialis (*M* = 174% ± 50, *p* < .001, *n* = 15), the medial gastrocnemius (*M* = 232% ± 111, *p* = .002, *n* = 12), the lateral gastrocnemius (*M* = 171% ± 73, *p* = .002, *n* = 15), the tibialis anterior (*M* = 210% ± 172, *p* = .021, *n* = 16), and the gluteus maximus (*M* = 180% ± 125, *p* = .027, *n* = 15) (Figure 1). The differences were not significant for the biceps femoris (*M* = 127% ± 105, *p* = .37, *n* = 12), the semitendinosus (*M* = 185% ± 141, *p* = .07, *n* = 13), or the gluteus medius (*M* = 139% ± 100, *p* = .18, *n* = 13) (Figure 1).

## Discussion

The purpose of the study was to examine EMG normalization difference with an MVIC in a drop jump movement. A desirable property of the MVIC would be that it elicits a maximum value close to the maximum value measured during the studied movement. Values greater than 100% therefore indicate that EMG amplitude recorded during the activity were greater than the maximum value recorded during the MVIC. In our measurements, this was statistically true for six of the nine examined muscles. The remaining three muscles activity maximum was still on average underestimated by the MVIC, but statistical significance likely obscured by substantial variability within the data. Overall, only 23% of our maximum activity recordings across all muscles were ≤100% MVIC. This is consistent with Suydam et al. (2017) who found that jumping elicited more amplitude in millivolts than from MVICs. What is a concern from a data interpretation standpoint is for the muscles that were significantly different from 100% activity maximum, the MVICs underestimated the maximal activity by 71%–140% on average, with 20% of our measures having an error of 260% or greater relative to MVIC.

We previously noted reasons that MVICs may underestimate maximum activity during drop jumps: the position the MVIC is recorded in (Contreras et al. 2015; Schwartz et al. 2020), contraction type (Enoka 1996; Grabiner and Owings 2002), participant motivation/encouragement (Mcnair et al. 1996), movement velocity (Ball and Scurr 2010), stretch shortening cycle augmentation (Trimble et al. 2000), and/or muscle crosstalk (Chapman et al. 2010). The current study cannot determine the specific contributions of each factor. For instance, the measures where significant underestimation occur likely have a high contribution from the participant’s effort/motivation during the MVIC, but there is no way to separate this from the reflexive augmentation of the EMG signal that occurs during the stretch shortening cycle.

Within the last 5 years of jumping, landing, and cutting research using EMG amplitude, we identified supramaximal activation compared to MVIC in 60.4% of studies using an MVIC to normalize EMG amplitude. We believe this may become significantly problematic when muscle activation is compared between different muscles, or an activation ratio is calculated (such as a quadriceps to hamstring activation). An MVIC which underestimates the activation potential of a muscle may be incorrectly interpreted as a muscle which is being activated to its maximum potential during a motion.

It is notable here that the underestimate of maximal muscle activity was both statistically significant and greater for the quadriceps muscles than the hamstring muscles. An often investigated variable is called the hamstring-to-quadriceps (H:Q) activation ratio. If MVICs underestimate hamstring activation less than quadriceps, these ratio calculations would be significantly different to what would be calculated if an activity maximum were used to normalize the data or if the peak muscle activity measured from MVICs truly represented maximal muscle activity. A recent example of the influence the MVIC underestimate can be found in work by Veeck et al. (2024). In that study, more than 50% participants elicited greater activity during a parallel squat (the exercise with the highest activity) than in an MVIC, but there was almost no instance where hamstring activity exceeded 100% MVIC (Veeck et al. 2024). Since quadriceps values exceeded 100% MVIC but hamstring values did not, the H:Q ratio is lowered across exercises not due to actual activation differences but due to the MVIC procedure being more effective in eliciting maximal activation from the hamstrings and not the quadriceps. It is clear that the normalization method has significant influence on data interpretation although its effect or limitations are not discussed. It would be interesting if the data normalization methods were compared within the context of Veeck et al. (2024) work.

What difference is acceptable between the maximum activity recording and MVIC may be dependent on the research question and study design. We find in our data, the normalization difference between an MVIC and actual activity maximums in drop jump landings is substantial and likely a contributing factor to the high observed variability in activation between participants and different muscle groups. Therefore, a between participant comparison or between muscle comparison using the values normalized to MVIC may not be appropriate with our data. It is important that researchers be aware of the possible difference between their MVIC and the maximum activity which can be elicited during the tested motion. This is important for correct interpretation of their EMG amplitude data in drop jump research.

## Conclusion

Maximal voluntary isometric contractions typically underestimate maximal muscle activity occurring in drop jumps. Values near 100%MVIC may therefore be interpreted as a maximum activation when in fact it may be less than half the true maximum activation depending on the muscle. Or in some outlier cases, less than 20% of true maximal activation. It may be necessary for researchers to quantify their normalization error when an MVIC is used in the EMG amplitude normalization processes and report possible impacts on the interpretation of the EMG amplitude signal and statistical outcomes. For instance, normalization error is greater for the knee extensor group than the knee flexor group impacting the interpretation of quadricep to hamstring activation ratios or values exceeding 200% MVIC may skew data, violating the assumption of normality. Researchers should carefully consider the impact that MVIC normalization may have on their data, and potentially report the difference compared to the maximum amplitude elicited during the motion under investigation. Researchers could also consider other normalization methods, such as normalizing to a sub-maximal contraction, if appropriate for their study design.
